# The relationship between dietary vitamin B1 intake and severe abdominal aortic calcification among the general population in the United States

**DOI:** 10.3389/fcvm.2024.1415151

**Published:** 2024-11-13

**Authors:** Hanbo Li, Ruihua Li, Changle Gong, Zhe Wu, Qiang Jia

**Affiliations:** ^1^ Department of Vascular Surgery, The First Affiliated Hospital of Shandong University of Traditional Chinese Medicine, Jinan, China; ^2^ Department of General Surgery, Vascular Surgery, Qilu Hospital of Shandong University, Jinan, China; ^3^ The First Clinical College, Shandong University of Traditional Chinese Medicine, Jinan, China; ^4^ Institute of Pharmaceutical Research, Shandong University of Traditional Chinese Medicine, Jinan, China

**Keywords:** dietary vitamin B1 intake, severe abdominal aortic calcification, NHANES, multivariable logistic regression, RCS

## Abstract

**Background:**

Vitamin B1 deficiency is closely associated with vascular system damage, but the relationship between dietary vitamin B1 intake and abdominal aortic calcification (AAC) remains unclear and warrants further investigation.

**Methods:**

2,640 participants from the National Health and Nutrition Examination Survey (NHANES) 2013–2014 were included in the study. Severe AAC was defined as Kauppila score >5. Multivariable logistic regression analysis and restricted cubic splines (RCS) were used to examine the relationship between dietary vitamin B1 and severe AAC.

**Results:**

The increase in dietary intake of vitamin B1 is significantly correlated with a decrease in the risk of severe AAC (OR: 0.601, 95% CI: 0.406, 0.892). Compared to the first quartile of dietary vitamin B1 intake, the fourth quartile had a significantly reduced risk of severe AAC (OR: 0.358, 95% CI: 0.172, 0.744). RCS indicated a decreasing trend in the risk of severe AAC with increasing dietary vitamin B1 intake.

**Conclusion:**

Our research findings indicate that the increase in dietary intake of vitamin B1 is significantly associated with a decrease in the risk of severe AAC. Thus, increasing dietary vitamin B1 intake appropriately may reduce the risk of severe AAC.

## Introduction

Abdominal aortic calcification (AAC) is a complex pathological process influenced by multiple factors, characterized by the pathological deposition of calcium phosphate crystals within the arterial intima (1). Research findings suggest that with advancing age, both the prevalence and severity of AAC tend to increase gradually (2). Currently known factors closely associated with the onset of AAC include age, chronic kidney disease, gender, diabetes, and hypertension, among others (3–6). Increasing evidence indicates that severe AAC serves as a risk factor for cardiovascular diseases (CVD) and is highly correlated with the rupture of atherosclerotic plaques, adverse cardiovascular events, and increased all-cause mortality risk (7, 8). Despite advancements, our understanding of the mechanisms underlying AAC remains incomplete, and effective preventive and treatment strategies are still lacking. Considering the significant correlation between severe vascular calcification and CVD as well as mortality rates (9), along with the challenges in treatment, further exploration of prevention and improvement methods for AAC is warranted.

Diet plays a crucial role in vascular health. Vitamin B1, as a water-soluble vitamin, is believed to be closely related to vascular health (10). Previous studies on the relationship between dietary vitamins and abdominal aortic calcification have mainly focused on dietary vitamins C, D, and K2 (11–14). However, there is still a lack of research on the relationship between dietary vitamin B1 intake and severe abdominal aortic calcification. Vitamin B1 plays an important role in regulating energy metabolism, maintaining endothelial cell function, and promoting nerve conduction (15). Previous studies have suggested an association between dietary vitamin B1 intake and increased risk of stroke and cardiovascular mortality (16). Despite the biological plausibility, the relationship between dietary vitamin B1 intake and severe AAC remains underexplored. Understanding this relationship is crucial, as it could inform dietary recommendations and public health strategies aimed at preventing or slowing the progression of AAC and its associated cardiovascular risks. Additionally, changing dietary habits as a non-invasive and cost-effective strategy for preventing severe abdominal aortic calcification is more acceptable and easier to implement for the general population.

National Health and Nutrition Examination Survey (NHANES) is a nationally representative survey that evaluates the health and nutritional status of the general United States population by gathering comprehensive data on diet, nutrition, and overall health. This study aims to evaluate the relationship between dietary vitamin B1 intake and AAC in the general population of the United States, utilizing data from NHANES 2013–2014. To fill the gap in research on the relationship between dietary vitamin B1 intake and severe abdominal aortic calcification.

## Methods

### Study population

**Figure 1:**
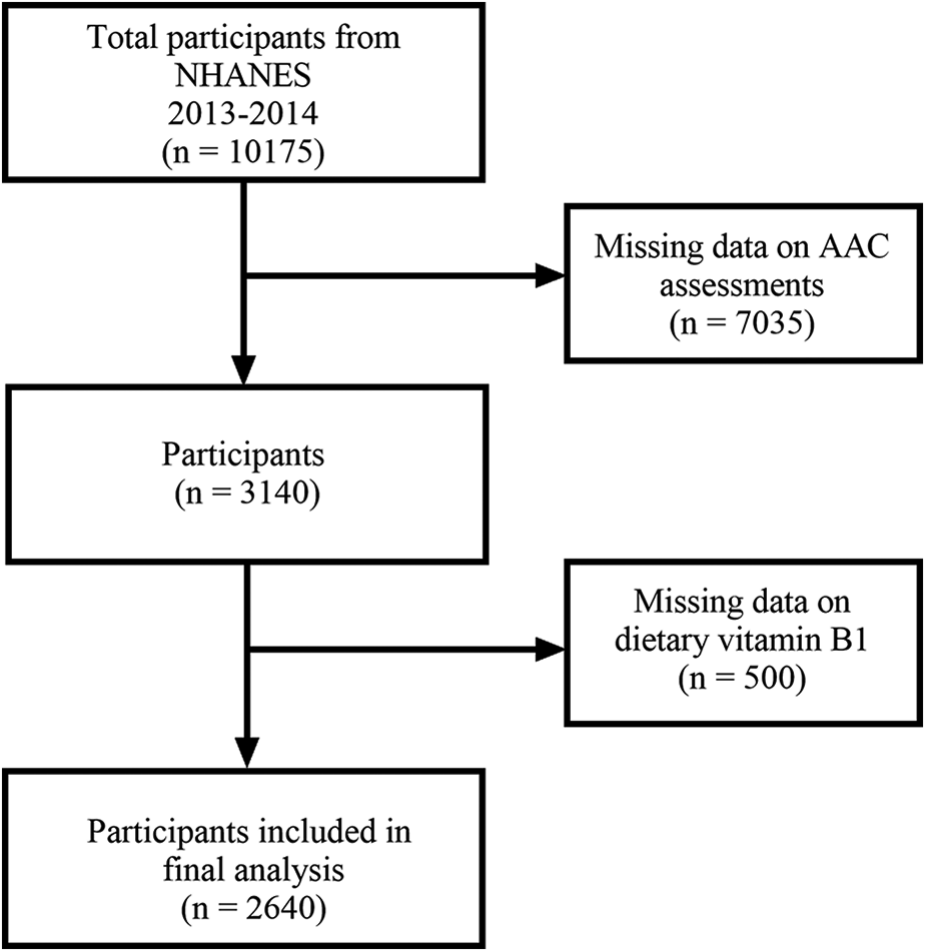
Flowchart depicting participant selection in the study.

NHANES is a cross-sectional study conducted on the general population of the United States aimed at investigating the nutritional and health-related information of the general population. We utilized relevant data on dietary vitamin B1 intake and AAC from NHANES 2013–2014. The 2013–2014 NHANES research protocol received approval from the National Center for Health Statistics. All subjects provided written informed consent forms. The deidentified data from the NHANES initiative is accessible to the general public at no cost. Under local regulations, this subsequent analysis does not necessitate additional authorization from the institutional review committee. A total of 10,175 participants were identified in NHANES 2011–2016. After excluding participants with missing AAC assessments (*n* = 7,035) and those lacking dietary vitamin B1 data (*n* = 500), a total of 2,640 participants were included in the final analysis (Figure 1).

Table 1 provides a detailed overview of the baseline characteristics of the 2,640 participants eligible for the study on dietary vitamin B1 and AAC, grouped by the presence or absence of severe AAC. Among them, there were 288 participants with severe AAC and 2,352 participants without severe AAC. The average age of the non-severe AAC group was 59.29 ± 0.27, while the average age of the severe AAC group was 71.03 ± 0.93. There were significant differences between the two groups in terms of age, total cholesterol levels, smoking history, alcohol consumption history, history of CVD, diabetes, and hypertension (*P* < 0.05).

**Table 1:** Population characteristics stratified by severe AAC.

Variable	Non-Severe AAC	Severe AAC	*P*-value
Age (years)	56.29 (0.27)	71.03 (0.93)	<0.01
Sex			0.56
Male	1,110 (47.96)	133 (44.91)	
Female	1,242 (52.04)	155 (55.09)	
Race			0.35
White	1,036 (69.67)	185 (75.10)	
Black	483 (10.75)	40 (7.36)	
Mexican American	312 (7.24)	25 (4.36)	
Other Race	521 (12.34)	38 (13.19)	
BMI (kg/m^2)^	28.71 (0.16)	27.30 (0.68)	0.07
Total cholesterol (mg/dL)	198.05 (1.35)	186.20 (3.35)	0.01
Smoke			0.01
Former	630 (26.39)	117 (41.09)	
Never	1,316 (57.46)	118 (42.98)	
Now	405 (16.15)	52 (15.93)	
Alcohol consumption			0.06
Former	447 (16.91)	91 (26.13)	
Never	336 (11.51)	32 (9.04)	
Mild	859 (40.19)	107 (43.78)	
Moderate	332 (18.16)	19 (8.43)	
Heavy	299 (13.24)	34 (12.63)	
CVD			<0.01
Yes	246 (9.64)	99 (31.92)	
No	2,105 (90.36)	189 (68.08)	
Diabetes			<0.01
Yes	485 (14.41)	122 (37.68)	
No	1,867 (85.59)	166 (62.32)	
Hypertension			<0.01
Yes	1,200 (48.01)	232 (77.70)	
No	1,152 (51.99)	56 (22.30)	

### Abdominal aortic calcification

AAC was defined using dual-energy x-ray absorptiometry. AAC was visually identified as diffuse white spots in the abdominal aorta. The severity of AAC was quantified using the Kauppila score. Severe AAC was defined as a Kauppila score >5.

### Dietary vitamin B1 intake

Dietary vitamin B1 intake was determined by recalling and calculating the food intake of participants over the past 24 h. The NHANES dietary interview procedures manual provided detailed instructions for this process.

### Covariates

The study included the following covariates: age, gender, race, BMI, smoking history (never, former, current), alcohol consumption history (never, former, mild, moderate, heavy), total cholesterol, CVD, hypertension, and diabetes status. In terms of smoking, never smoking is defined as smoking less than 100 cigarettes in one's lifetime. Former smoking was defined as having smoked more than 100 cigarettes in one's lifetime but now having completely quit smoking. Now smoking is defined as having smoked more than 100 cigarettes in one's lifetime and currently smoking on some days or every day. In terms of alcohol consumption, never alcohol consumption is defined as drinking less than 12 glasses in one's lifetime. Former alcohol consumption is defined as drinking at least 12 times within a year and not drinking in the previous year, or not drinking in the previous year but drinking at least 12 glasses throughout one's life. Mild alcohol consumption was defined as 0–2 drinks/day for males and 0–1 drinks/day for females. Moderate alcohol consumption was defined as 3 drinks/day for males and 2 drinks/day for females, or binge drinking at least 2 times a month and less than 5 times. Heavy alcohol consumption is defined as men drinking at least 4 drinks per day, women drinking at least 3 drinks per day, or binge drinking at least 5 times a month. CVD was determined through a questionnaire. Hypertension was defined as an average systolic blood pressure ≥140 mmHg and/or an average diastolic blood pressure ≥90 mmHg, diagnosed by a doctor, or the use of antihypertensive drugs. Diabetes was defined as a random blood glucose level ≥11.1 mmol/L, fasting blood glucose ≥7 mmol/L, glycosylated hemoglobin ≥6.5%, two-hour OGTT blood glucose ≥11.1 mmol/L, diagnosed by a doctor, or the use of hypoglycemic drugs.

### Statistical analysis

Statistical analysis was performed using R Studio (version 4.2.1) with weighted analysis based on dietary factors. Weighted Student's *t*-test, Mann Whitney *U*-test, and Chi-squared test were used to compare the differences between the two groups. Continuous variables were presented as mean (standard error), while categorical variables were presented as numbers (weighted percentages). We divide the intake of vitamin B1 into quartiles, Q1 < 1.09 mg, 1.09 mg ≤ Q2 < 1.43 mg, 1.43 mg ≤ Q2 < 1.89 mg, Q2 ≥ 1.89 mg. The OR and 95% CI are calculated in the “survey” R package. The relationship between dietary vitamin B1 and AAC was explored using multivariable logistic regression. We used an restricted cubic splines (RCS) model with four knots to further explore the association between dietary vitamin B1 and AAC after adjusting for all covariates. We selected the median of dietary vitamin B1 intake as the reference value (1.42 mg). Subgroup analyses were conducted based on age, gender, smoking, alcohol consumption, hypertension, CVD, and diabetes status to investigate the relationship between dietary vitamin B1 intake and severe AAC. The *P* for interaction is obtained using a likelihood ratio test.

## Results

### Dietary vitamin B1 intake and severe AAC

To validate the relationship between dietary vitamin B1 intake and AAC, we conducted a multivariable logistic regression analysis. In the unadjusted model (OR: 0.662, 95% CI: 0.511, 0.857), after adjusting for age, gender, race (OR: 0.583, 95% CI: 0.370, 0.917), and adjusting for all covariates (OR: 0.601, 95% CI: 0.406, 0.892), the increase in dietary intake of vitamin B1 is significantly correlated with a decrease in the risk of severe AAC. After full adjustment for covariates, compared to the first quartile of dietary vitamin B1 intake, the fourth quartile showed a significantly reduced risk of severe AAC (OR: 0.358, 95% CI: 0.172, 0.744) (Table 2).

**Table 2:** Relationship between dietary vitamin B1 and severe AAC.

Result	Model 1		Model 2		Model 3	
	OR (95% CI)	*P*-value	OR (95% CI)	*P*-value	OR (95% CI)	*P*-value
Dietary vitamin B1 and severe AAC	0.662 (0.511, 0.857)	0.004	0.583 (0.370, 0.917)	0.025	0.601 (0.406, 0.892)	0.015
Q1	ref	ref	ref	ref	ref	ref
Q2	0.655 (0.275, 1.562)	0.310	0.619 (0.231, 1.660)	0.288	0.716 (0.285, 1.795)	0.450
Q3	0.840 (0.556, 1.268)	0.375	0.658 (0.402, 1.077)	0.084	0.741 (0.466, 1.179)	0.189
Q4	0.426 (0.259, 0.701)	0.003	0.354 (0.153, 0.820)	0.022	0.358 (0.172, 0.744)	0.009

Adjusted variables: Model 1: unadjusted. Model 2: age, sex, race. Model 3: age, sex, race, BMI, smoking history, alcohol consumption history, total cholesterol, cardiovascular diseases, hypertension, and diabetes.

OR, odds ratio; CI, confidence interval.

### Restricted cubic splines

**Figure 2:**
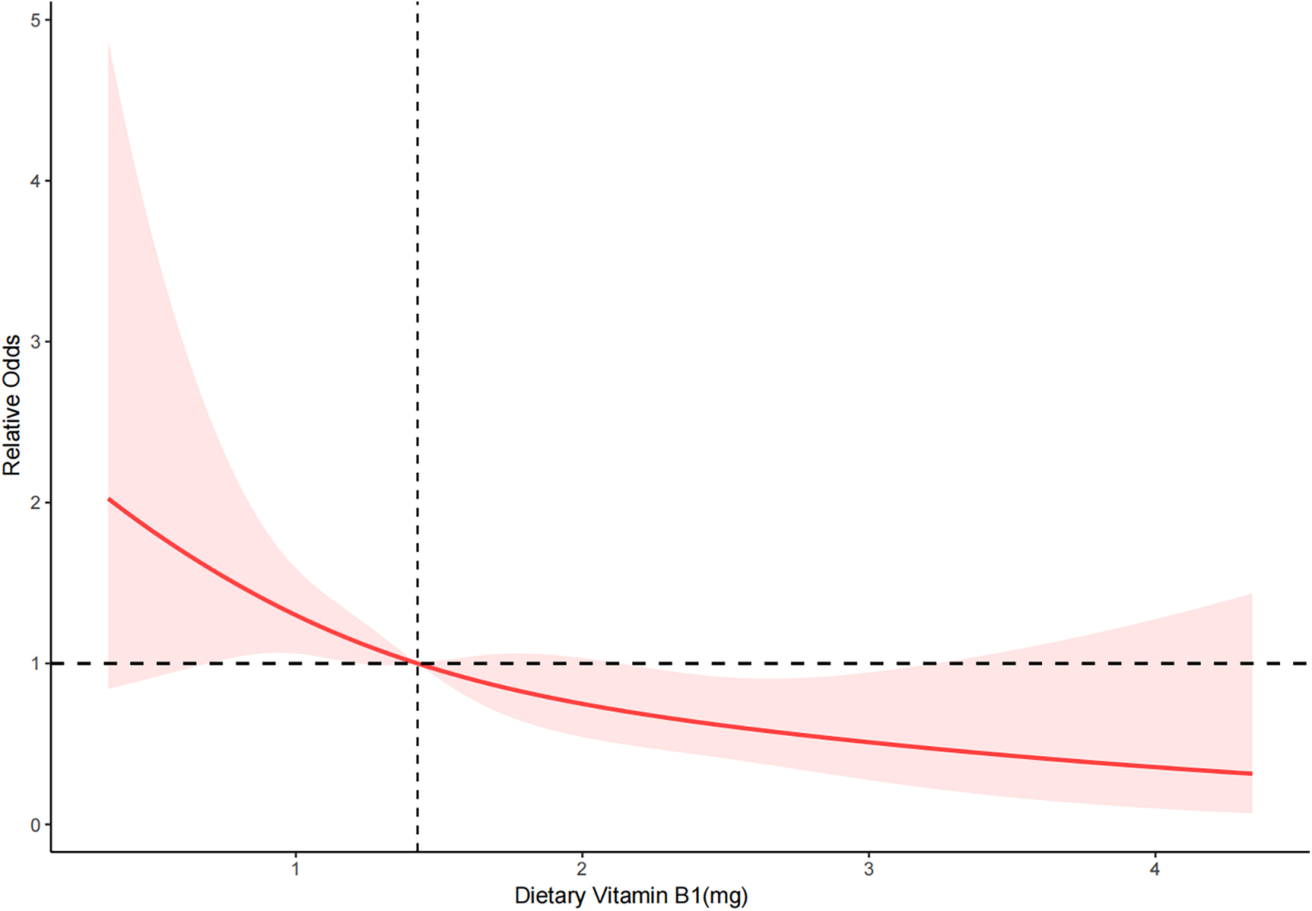
RCS of dietary vitamin B1 and severe AAC. The red solid line represents the smooth curve fitting between variables. The light red area represents the fitted 95% confidence interval.

RCS depicted the dose-response relationship between dietary vitamin B1 intake and severe AAC. The results showed a linear negative correlation between dietary vitamin B1 intake and the risk of severe AAC (*P* for overall = 0.003 < 0.05, *P* for nonlinearity = 0.852 > 0.05) (Figure 2).

### Subgroup analysis

A subgroup analysis was conducted to examine the relationship between dietary vitamin B1 intake and severe AAC based on age, gender, smoking status, alcohol consumption, presence of diabetes, hypertension, and CVD. All analyses were adjusted for all covariates, except for the stratifying variables (Table 3). The results indicate that the relationship between dietary vitamin B1 intake and severe AAC remains significant in participants aged 60 years and younger, females, current smokers, and those without CVD and diabetes. Interaction analysis revealed that the negative correlation between dietary vitamin B1 intake and severe AAC was more pronounced in participants aged 60 years and younger, females, and current smokers (*P* for interaction <0.05). Additionally, all subgroup analysis results consistently showed the increase in dietary intake of vitamin B1 is significantly correlated with a decrease in the risk of severe AAC, indicating robust findings across subgroups.

**Table 3:** Subgroup analysis and interaction test of dietary vitamin B1 and severe AAC.

Variable	OR (95% CI)	*P*-value	*P* for interaction
Age			0.025
>60 years old	0.730 (0.461, 1.154)	0.164	
≤60 years old	0.195 (0.049, 0.782)	0.024	
Sex			0.026
Male	0.774 (0.484, 1.239)	0.264	
Female	0.375 (0.234, 0.602)	<0.001	
Smoke			0.023
Former	0.761 (0.353, 1.642)	0.461	
Never	0.699 (0.408, 1.197)	0.177	
Now	0.188 (0.072, 0.489)	0.002	
Alcohol consumption			0.433
Former	0.905 (0.342, 2.394)	0.701	
Never	0.972 (0.187, 5.059)	0.948	
Mild	0.627 (0.137, 2.881)	0.319	
Moderate	0.173 (0.001, 29.355)	0.279	
Heavy	0.566 (0.066, 4.884)	0.373	
CVD			0.343
Yes	0.853 (0.479, 1.521)	0.567	
No	0.568 (0.381, 0.846)	0.008	
Hypertension			0.74
Yes	0.617 (0.359, 1.061)	0.077	
No	0.551 (0.239, 1.275)	0.151	
Diabetes			0.255
Yes	0.805 (0.346, 1.874)	0.592	
No	0.480 (0.319, 0.722)	0.002	

OR, odds ratio; CI, confidence interval.

## Discussion

This study aimed to investigate the relationship between dietary vitamin B1 intake and severe AAC in the general population of the United States. Our study results demonstrate that the increase in dietary intake of vitamin B1 is significantly associated with a decrease in the risk of severe AAC. Insufficient dietary vitamin B1 intake may increase the risk of severe AAC. For populations with low dietary intake of vitamin B1, it is recommended to increase the intake of vitamin B1 to reduce the risk of severe AAC. To our knowledge, this is the first study exploring the relationship between dietary vitamin B1 intake and AAC in the general population.

In fact, epidemiological research on AAC is limited. Existing studies have shown that the incidence of AAC is very high. The Framingham Heart Study results indicate that the age of onset of AAC is mainly concentrated between 45 and 65 years old. More than 90% of people aged 65 and above suffer from varying degrees of AAC (2). The Multi-Ethnic Study of Atherosclerosis found that 80% of non-Hispanic Whites, 68% of Hispanic Americans and 63% of African Americans had AAC through the study of 1,957 participants with an average age of 65 years (17). However, more and more evidence suggests that AAC can be treated or even reversed in terms of calcification degree. This prompts people to search for possible treatment methods (18).

Vascular calcification is a process where vascular smooth muscle cells undergo transdifferentiation into osteoblast-like cells under various pathological factors, mediating the abnormal deposition of calcium salts in the vascular wall (19). The pathophysiological mechanisms of this disease are extraordinarily complex and remain incompletely understood, involving cellular osteogenic differentiation, inflammation, oxidative stress, apoptosis, and autophagy (20). Among these, oxidative stress and inflammation play crucial roles in the development of vascular calcification (21). Studies have shown that inflammation is associated with osteogenic activity in the cardiovascular system and vascular calcification. Oxidative stress can activate signaling molecules in the inflammation pathway, such as nuclear transcription factor *κ*B, and a series of inflammatory mediators, such as tumor necrosis factor-α, interleukin-1 (22–24). The release of these inflammatory mediators and activation of inflammatory cells trigger an inflammatory response in the vascular wall, accelerating endothelial cell damage and proliferation, ultimately promoting calcium salt deposition in the vascular wall and the formation of vascular calcification.

Vitamin B1, also known as thiamine, is the first B-vitamin discovered (25). It is a complex organic molecule that acts as a coenzyme in various reactions of glycolysis and the tricarboxylic acid cycle. The primary physiological function of vitamin B1 is to participate in energy metabolism, while also playing important roles in maintaining the normal function of nerves, muscles, especially cardiac muscles, as well as regulating normal appetite, gastrointestinal motility, and digestive secretion (26). In the past, vitamin B1 has been shown to improve endothelial and smooth muscle cell function by reducing the formation of glycolytic metabolism products and inhibiting the proliferation of vascular smooth muscle cells, and it has protective effects against glucose and insulin-mediated proliferation of human vascular smooth muscle cells (27–29). Additionally, vitamin B1 can reduce the degree of oxidative stress in the body, inhibit the production of free radicals, thus protecting endothelial cells and other cells in the vascular wall from oxidative damage (30). Despite some preliminary research findings, there is currently no literature reporting the effect of vitamin B1 on AAC. Our study results suggest a significant correlation between dietary vitamin B1 intake and AAC, indicating that increasing dietary vitamin B1 intake may have a positive effect on reducing the risk of AAC. However, this association and its specific mechanisms require further investigation.

We must acknowledge the limitations of this study. One of the major limiting factors is that this is a cross-sectional study, and therefore, it cannot determine the specific mechanisms underlying the correlation between dietary vitamin B1 intake and AAC. Additionally, the study population included individuals aged 40 and above, so further exploration is needed for the relationship between dietary vitamin B1 intake and AAC in adults under 40 years of age. The study population was limited to the general population in the United States, so caution should be exercised when generalizing these results to other populations. In addition, there is a lack of more recent data on abdominal aortic calcification. The data on abdominal aortic calcification is only available in NHANES 2013–2014, which may result in potential selection bias.

## Conclusion

Our research findings indicate that the increase in dietary intake of vitamin B1 is significantly associated with a decrease in the risk of severe AAC. Insufficient dietary vitamin B1 intake may increase the risk of severe AAC.

## Data Availability

The raw data supporting the conclusions of this article will be made available by the authors, without undue reservation.
